# Better models by discarding data?

**DOI:** 10.1107/S0907444913001121

**Published:** 2013-06-15

**Authors:** K. Diederichs, P. A. Karplus

**Affiliations:** a Faculty of Biology, University of Konstanz, M647, 78457 Konstanz, Germany; b Department of Biochemistry and Biophysics, Oregon State University, Corvallis, OR 97331, USA

**Keywords:** *R* value, correlation coefficient, data quality, model quality, outlier rejection

## Abstract

In macromolecular X-ray crystallography, typical data sets have substantial multiplicity. This can be used to calculate the consistency of repeated measurements and thereby assess data quality. Recently, the properties of a correlation coefficient, CC_1/2_, that can be used for this purpose were characterized and it was shown that CC_1/2_ has superior properties compared with ‘merging’ *R* values. A derived quantity, CC*, links data and model quality. Using experimental data sets, the behaviour of CC_1/2_ and the more conventional indicators were compared in two situations of practical importance: merging data sets from different crystals and selectively rejecting weak observations or (merged) unique reflections from a data set. In these situations controlled ‘paired-refinement’ tests show that even though discarding the weaker data leads to improvements in the merging *R* values, the refined models based on these data are of lower quality. These results show the folly of such data-filtering practices aimed at improving the merging *R* values. Interestingly, in all of these tests CC_1/2_ is the one data-quality indicator for which the behaviour accurately reflects which of the alternative data-handling strategies results in the best-quality refined model. Its properties in the presence of systematic error are documented and discussed.

## Abstract 2

Making the most of hard-won data in protein crystallography: to keep or not to keep, that is the question.

## 1. Introduction

Since the number of reflections in a crystallographic experiment is high, indicators of aggregated statistical properties are needed. For decades, the ‘merging’ *R* value, 
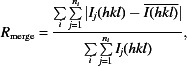
has been used almost exclusively for this purpose. This normalized linear residual was defined (Arndt *et al.*, 1968 ▶) *ad hoc* to measure the consistency of measurements made with the first two-dimensional detectors by utilizing the multiplicity (also called redundancy) of observations of the unique reflections. The formula sums the absolute deviations of intensities of *n*_*i*_ observations of unique reflections from their averages and normalizes using the sum of the intensities. As a relative measure of deviation, it can be calculated as an overall quantity for a data set, but also as a function of resolution. Later, it was shown (Diederichs & Karplus, 1997 ▶) that each term of the numerator has to be modified by a factor of [*n*_*i*_/(*n*_*i*_ − 1)]^1/2^ to give a result that is independent of the average multiplicity. The resulting quantity is called *R*_meas_ (or the redundancy-independent merging *R*, *R*_r.i.m._; Weiss, 2001 ▶) and reports on the consistency of the measured observations. It was also realised that a higher multiplicity of observations results in the merged data being of higher quality than the individual measurements, so a distinct statistic, *R*_mrgd_, was introduced to assess the **merged** data quality (Diederichs & Karplus, 1997 ▶). Thereafter, it was shown that the quality of the merged data could also be estimated by introducing an additional factor of 1/*n*_*i*_^1/2^ into each term of the *R*_meas_ numerator (Weiss, 2001 ▶), as this accounts for the expected increase in accuracy associated with averaging *n_i_* measurements. The resulting quantity is called *R*_p.i.m._ (the precision-indicating merging *R*; Weiss, 2001 ▶).

Recently, we (Karplus & Diederichs, 2012 ▶) and Evans (2011 ▶) have suggested that the Pearson correlation coefficient of two ‘half’ data sets (*i.e.* each derived by averaging half of the observations for a given reflection) might be better suited than merging *R* factors for assessing data quality. In our work, we designated this quantity CC_1/2_ and conclusively showed that in many ways it has a better statistical foundation than merging *R* values (Karplus & Diederichs, 2012 ▶) and that in particular it provides a direct assessment of the relative proportions to which signal and noise contribute to the variation in the data in a given resolution shell. Furthermore, we introduced a quantity termed CC*, defined as
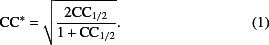



**Figure 1:**
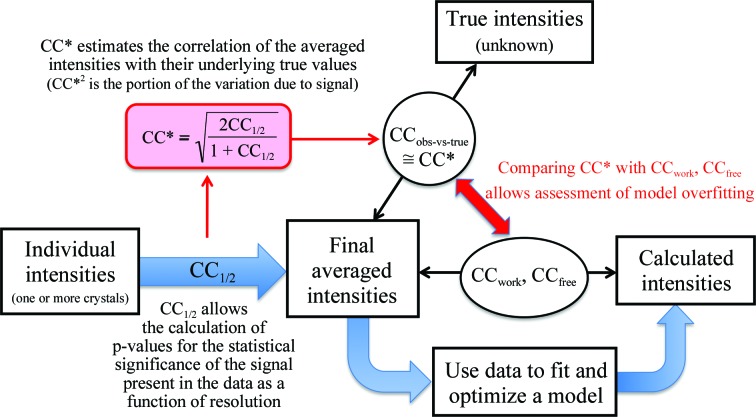
Scheme documenting the relationships of correlation coefficients calculated between squared observed and calculated amplitudes. This figure was adapted from Diederichs & Karplus (2013 ▶).

CC* (with an associated uncertainty) is an experimental estimate of what could be called CC_true_, the correlation of the final merged data with the underlying true values of these quantities. CC* is thus an upper limit for CC_work_ and CC_free_ from a properly refined model, where the latter are correlation coefficients between intensities calculated from the model and those obtained from the experiment. CC* is limiting because if the intensities calculated from a model match the experimental data better than the (unknown) true intensities do, this implies that the model is overfitted, fitting not just the signal but also the noise that is in the data. The relationships between these correlation coefficients are schematically summarized in Fig. 1 ▶.

Any scientific experiment entails data processing, which can include outlier detection and rejection. In crystallography, common practices include systematically rejecting certain subsets of data in order to ‘improve’ the resulting merged data set. The kinds of data that are sometimes rejected are whole data sets, high-resolution shells, unique reflections in the final reduced data set and single observations before merging or combinations thereof. While these practices do serve to improve the merging *R*-value statistics associated with the data, as far as we are aware little effort has been made to assess how these procedures impact the quality of the model that is produced by refinement against the data, which in our view is the single outcome that matters.

The only such study of which we are aware is our recent work using a novel ‘paired-refinement’ strategy to show that the inclusion of often-rejected weak data in high-resolution shells significantly improves the quality of the refined models (Karplus & Diederichs, 2012 ▶). Paired refinement means that a starting model is refined using the same refinement protocol against both a full data set and an (in some way) truncated version of the full data set. The resulting two models are then compared in terms of *R* values (*R*_work_, *R*_free_) to judge which model is better. Importantly, the comparison of *R* values is only meaningful when the truncated data set is also used to calculate *R*_work_ and *R*_free_ of the model that was refined against the full data set.

For our test cases, paired refinements indicated that including data to the resolution at which CC_1/2_ was in the range 0.1–0.2 led to an improved model, even though at these resolutions the traditionally used statistics were well beyond the conventional limits (the limiting signal-to-noise ratios were near 0.3–0.6 and *R*_meas_ was in excess of 300%). Interestingly, this CC_1/2_ range is a reasonable match to the value of 0.143 which was proposed in the field of electron microscopy for a quantity (FSC; Fourier shell correlation) related to CC_1/2_ as an appropriate limit for discarding data because they correspond to a CC* value of 0.5 (Rosenthal & Henderson, 2003 ▶). Although this value indeed seems to be a reasonable cutoff for X-ray data for the test cases we studied, we resist generalizing this point and suggest that the high-resolution cutoff is in general better decided using the ‘paired-refinement’ strategy (Karplus & Diederichs, 2012 ▶). The latter approach has two main advantages: firstly, it allows for the possibility that individual structures or data sets may behave differently, and secondly, it allows for the possibility that as refinement programs improve they may be able to more fully extract structural information from weak data.

Given the paired-refinement technique and the novel correlation coefficient-based data-quality indicators, it is now possible to systematically investigate the impacts of other data-selection practices mentioned above. Here, we document further properties of CC_1/2_ and CC* and also explore how the various debatable ‘data-filtering’ procedures impact these new and the conventional *R*-value-based statistics, as well as model quality.

## 2. Materials and methods

### 2.1. Data sets

Crystals of cysteine dioxygenase (CDO) were grown and soaked as described previously (Simmons *et al.*, 2008 ▶). Data frames were collected on beamline 5.0.1 at the Advanced Light Source.

The data frames were processed and scaled with *XDS* (v. December 6, 2010; Kabsch, 2010*a* ▶,*b* ▶) and data sets were merged with *XSCALE* using default parameters. The space group is *P*4_3_2_1_2, with average unit-cell parameters *a* = *b* = 57.5, *c* = 122.2 Å.

### 2.2. Quality indicators

In addition to data-quality *R* values, it is conventional to quantify the signal-to-noise ratio of the merged intensities, given as 〈*I*/σ〉. We used a custom program *HIRESCUT* to evaluate *R*_meas_, 〈*I*/σ〉, CC_1/2_ and CC* as a function of resolution, a feature that has since been merged into *XDS* and *XSCALE*. Furthermore, we added functionality to *HIRESCUT* that allows the rejection of single observations or unique reflections according to their *I*/σ ratio.

The *CCP*4 (Winn *et al.*, 2011 ▶) program *SFTOOLS* was used to obtain correlation coefficients between experimental intensities and *F* ^2^_calc_ from the model.

### 2.3. Refinement

To obtain a model suitable for refinement, we used the PDB entry representing a related structure (PDB entry 2b5h; Simmons *et al.*, 2006 ▶); the *S*-cysteine-persulfenate ligand was added guided by a difference Fourier map. H-atom positions were constructed and the resulting PDB file was refined in *phenix.refine* (v.1.7.1; Adams *et al.*, 2010 ▶) using default parameters for the weights and number of macrocycles and updating the solvent model in every cycle.

## 3. Results and discussion

The main goal of this work is to consider three common questions that arise as part of data reduction and explore how the new correlation-coefficient-based data-quality measures behave and what the paired-refinement strategy indicates about the choices that will deliver the best refined models. The first of these scenarios is that of having a strong data set and a weaker data set, and we ask whether it is wise to merge the two data sets or to just use the stronger one. The other two scenarios have to do with practices that are not considered good practice by experts but are sometimes used to improve data-reduction statistics by deleting selected weak reflections.

### 3.1. Can strong data be improved by merging with a weaker data set?

This set of analyses uses three data sets (CDO3, CDO4 and CDO5, each corresponding to 90° of rotation) collected from different crystals that were grown and handled equivalently. According to all data-quality indicators [CC_1/2_, *R*_meas_ and 〈*I*/σ(*I*)〉] CDO3 is the best data set (Table 1 ▶), with CDO4 and CDO5 being similar to each other and of lower quality than CDO3. These data sets were merged in all possible combinations, and refinement in *phenix.refine*, both isotropically and anisotropically, was carried out against the resulting data sets using the same test set of reflections and the same high-resolution limit of 1.57 Å.

Using Table 1 ▶, we can investigate the question of how strongly *R*_meas_, CC_1/2_ and 〈*I*/σ(*I*)〉 are associated with the quality of the resulting model. We find examples of both improvement and deterioration of the merged data set depending on its constituent data sets.

The increased value of *R*_meas_, if taken as an indicator of data quality, would suggest that merging of CDO3 (*R*_work_/*R*_free_ = 0.186/ 0.219) with either CDO4 or CDO5 should significantly decrease the quality of the resulting data set, but in fact, based on the overall *R*_work_/*R*_free_, the model quality slightly decreases for CDO3+4 (*R*_work_/*R*_free_ = 0.185/0.221) but slightly improves for CDO3+5 (*R*_work_/*R*_free_ = 0.185/0.216). However, the comparison of model *R* values in this way is not really meaningful, since they are calculated against different data sets. This problem is overcome by the paired-refinement technique (Table 2 ▶), which unambiguously confirms that the model obtained by refinement against CDO3+5 fits data set CDO3 better than the original model obtained by refinement against CDO3. Conversely, the paired-refinement technique confirms that refinement against a merged CDO3+4 data set does not produce a model that better fits CDO3 than the original model.

In both cases, CC_1/2_/CC* correctly predict this result: the value of CC* in the highest resolution shell is increased relative to CDO3 for the CDO3+5 data set but not for the CDO3+4 data set.

In contrast, in the case of 〈*I*/σ(*I*)〉 the overall value decreases but the value in the highest resolution shell increases in both cases, so the influence of data set merging upon model quality is difficult to anticipate.

Merging of the two weak CDO4 and CDO5 data sets yields a better data set, as revealed by the decreased *R*_free_ (*R*_work_/*R*_free_ = 0.199/0.237) of the model refined against it. However, this model only fits CDO5 better than the original model refined against CDO5; it does not fit the CDO4 data set better despite the increased CC*. The explanation in this case is most likely that CDO4 is less complete, in particular in the highest resolution shell, than CDO3 and CDO5. Therefore, the caveat mentioned above, namely that comparison of crystallographic indicators should be performed against the same data, applies. In addition, slight non-isomorphism between CDO4 *versus* CDO3 and CDO5 is conceivable. In this case in particular, it is important to use the paired-refinement technique.

We note that in all cases the isotropic CC_work_ (Table 1 ▶) is significantly worse than the anisotropic value and that the anisotropic *R*_free_ is lower by 0.5–1.3% than the isotropic *R*_free_ (Table 1 ▶), which indicates that there is some justification for anisotropic refinement. However, we decided not to show the detailed results of anisotropic refinement since it is not completely justified: compared with isotropic refinement, anisotropic refinement reduced *R*_work_ by about 3%, thus widening the *R*_free_–*R*_work_ gap; likewise, a wider CC_work_–CC_free_ gap is observed for anisotropic refinement than for isotropic refinement. This is consistent with the observation that the anisotropic CC_work_ reaches or slightly exceeds CC*, indicating overfitting.

In summary, *R*_meas_ is not a useful indicator of merged data-set quality, whereas CC_1/2_, and to a lesser extent 〈*I*/σ(*I*)〉, correctly indicate the direction of quality change upon merging. CC* is a meaningful upper limit of CC_work_; CC_work_ of isotropic models is found to be significantly lower than CC*, whereas CC_work_ of anisotropic models is slightly higher than CC*. The latter finding also suggests that sphericity restraints in anisotropic refinement are a desirable feature of a refinement program; currently, only *SHELXL* (Sheldrick, 2008 ▶) and *REFMAC* (Murshudov *et al.*, 2011 ▶) support this.

### 3.2. Does discarding weak observations to improve conventional data-quality statistics actually lead to better models?

**Figure 2:**
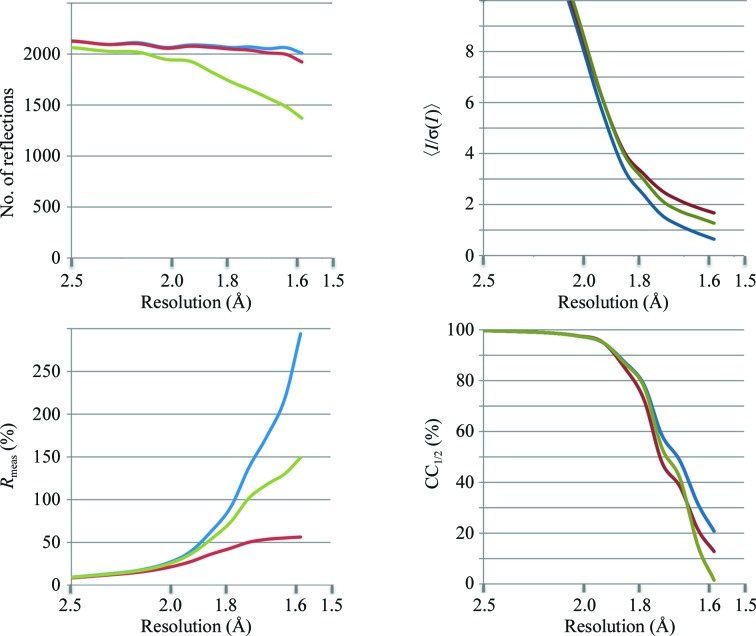
Data statistics for CDO3 (blue), CDO3b (green) and CDO3c (red).

It is obvious that by discarding the weakest data it is possible to create a data set with better conventional statistics at high resolution [*i.e.* lower *R*_meas_ and higher 〈*I*/σ(*I*)〉], so that a higher resolution cutoff appears to be justified. While we did not find publications about these practices, we know from discussion with students and colleagues that they are being applied, in particular to prevent possible criticism by reviewers. To assess the impact of such ‘data-filtering’ practices on the CC_1/2_ statistic and on model quality, we reprocessed the CDO3 data set to reject either all negative unique reflections (data set CDO3b) or all negative observations (data set CDO3c). The high-resolution statistics for the three data sets (Fig. 2 ▶) shows a few interesting features. Firstly, as expected, both CDO3b and CDO3c have much lower *R*_meas_ and higher 〈*I*/σ(*I*)〉 in the high-resolution bins. Secondly, more reflections are rejected in CDO3b, which makes sense because any reflections having at least a single positive observation will be included in CDO3c, while even reflections with positive observations will be deleted from CDO3b whenever the positive observations are more than offset by negative observations of the same reflection. Thirdly, the high-resolution CC_1/2_ values of both CDO3b and CDO3c are lower than those of CDO3, with CDO3b showing the greater decrease.

To assess the relative quality of the models resulting from refinements against these three data sets applying the paired-refinement strategy, the same starting model was refined against each data set and the resulting models were used, without further refinement, to obtain *R*_work_ and *R*_free_ against the other two data sets. These *R* values (Table 3 ▶) show that the model resulting from refinement against all reflections (data set CDO3) is unambiguously the best model, giving both the lowest *R*_free_ and the least overfitting (as judged from the reduced *R*_free_–*R*_work_ gap) with all three data sets. Furthermore, the model refined against data set CDO3b is consistently better than that resulting from refinement against CDO3c, even though data set CDO3b has fewer unique reflections than data set CDO3c. We conclude that data ‘massaging’ or ‘filtering’ by rejecting negative unique reflections, or – even worse – negative observations, with the purpose of enhancing *R*_meas_ or 〈*I*/σ(*I*)〉 values is counterproductive and leads to worse models. This conclusion is entirely consistent with the concept that the inclusion of weak data (even so weak as to be negative), when appropriately weighted, improves the resulting model and that they should not be discarded.

**Figure 3:**
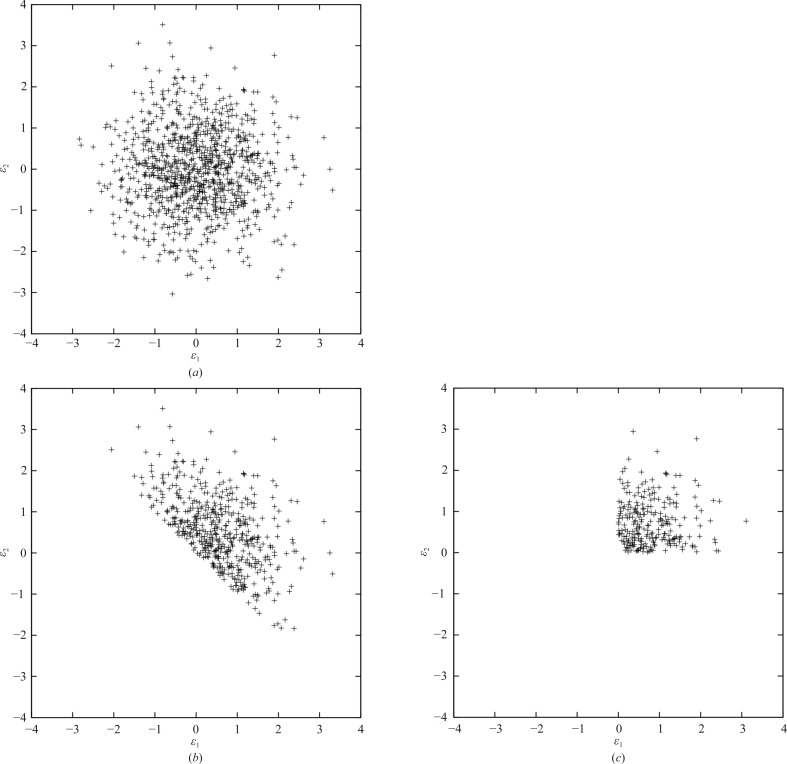
Example demonstrating the possibility of negative CC_1/2_ when rejecting reflections with negative intensities from a data set. The plots show ∊_1_ *versus* ∊_2_ for simulated data having Gaussian noise and no signal (τ = 0). (*a*) 1000 unique reflections, each represented by two observations; no rejections. The correlation of ∊_1_ and ∊_2_ is near zero. (*b*) From the 1000 unique reflections, those with negative intensity (∊_1_ + ∊_2_ < 0) were rejected. The resulting correlation between ∊_1_ and ∊_2_ is about −0.47. (*c*) From the 1000 unique reflections, those with negative ∊_1_ or negative ∊_2_ were rejected, also resulting in positive (merged) intensity. The resulting correlation between ∊_1_ and ∊_2_ is near zero.

In contrast to the behaviour of *R*_meas_ and 〈*I*/σ(*I*)〉, the CC_1/2_ values at high resolution of the CDO3b and CDO3c data sets actually decrease, paralleling the changes in model quality even though CC_1/2_ might be expected to also increase given that the remaining data are stronger. As is illustrated in Fig. 3 ▶, the data-filtering practices are not producing a typical data set with a higher signal-to-noise ratio, but are introducing large systematic errors into the data by skewing the distribution of reflection intensities from what would be expected for a data set that has a certain level of signal and random errors. In the next section, we present an analysis of how systematic errors such as these can influence the CC_1/2_ and CC* values.

### 3.3. Theory of the impact of systematic errors on CC_1/2_ and CC*

Here, we use the terminology and definitions of the work that introduced CC_1/2_ (Karplus & Diederichs, 2012 ▶). To calculate the intra-data-set correlation coefficient CC_1/2_, the measurements belonging to each unique reflection of the experimental data set are randomly assigned to two half data sets and the observations belonging to each half data set are averaged to give *I*_1_ and *I*_2_, respectively. We observe that by choosing observations randomly, none of the two half data sets is preferred in any way; thus, their variances σ^2^_∊1_ and σ^2^_∊2_ are the same (σ_∊_^2^). We may thus define *J* − 〈*J*〉 = τ for ‘true’ measurements with mean zero and variance σ_τ_^2^, ∊_1_ as the errors in random half data set 1, with an expectation value of zero and variance σ_∊_^2^, and ∊_2_ as the errors in random half data set 2, with an expectation value of zero and variance σ_∊_^2^.

CC* as given by (1) is an estimate of CC_true_, the correlation between the arithmetic average of the half data set intensities *I*_1_ and *I*_2_ and the true intensities *J*. This estimate should be accurate since no approximations are involved in deriving (1). When deriving (1), we assumed that τ, ∊_1_ and ∊_2_ are mutually independent. However, when systematic errors in the measurement or data processing are present τ, ∊_1_ and ∊_2_ may no longer be independent of each other.

An example of systematic error that leaves τ, ∊_1_ nd ∊_2_ mutually independent is the case of an error in a data-processing program that results in a positive offset to all intensities. This is clearly a systematic error, but it does not affect τ, ∊_1_ and ∊_2_ (since any offset is subtracted when constructing τ, ∊_1_ and ∊_2_, which have an expectation value of zero) and thus has no influence on CC_1/2_ or CC*. This type of error would, however, lower *R*_merge_ but increase *R*_work_/*R*_free_. In this ‘Gedankenexperiment’, data and model *R* values are thus anticorrelated, whereas a CC-based data-quality indicator is unchanged. A simple way to detect such a problem would be to monitor the scale factors between *I*_obs_ and *I*_calc_ [note that comparing *I*_obs_ and *I*_calc_ rather than *F*_obs_ and* F*_calc_ avoids artifacts introduced by the French–Wilson (French & Wilson, 1978 ▶) procedure for converting intensities to amplitudes].

Three conceptually simple examples for systematic errors that **do** invalidate one or more of the assumptions of independence are the following.

(i) Owing to an error in space-group assignment that can, for instance, occur in special cases of pseudosymmetric translational symmetry, only every second reflection is processed; the missed reflections are wrongly assigned an intensity of zero. If we consider all reflections, including the missed ones, ∊_1_ and ∊_2_ are (positively) correlated (*i.e.* non-independent); in particular, they are negative for the missed reflections. τ is (negatively) correlated with ∊_1_ and ∊_2_.
(ii) Owing to overflow or nonlinearity of the detector hardware, the intensity of strong reflections may be underestimated. Also in this case the true signal τ is (negatively) correlated with ∊_1_ and ∊_2_, and ∊_1_ and ∊_2_ are (positively) correlated with each other (*e.g.* if one of them is negative, the other is often negative as well).
(iii) Inadequate scaling or radiation damage may yield intensities that are too low or too high for half of the observations of each unique reflection, respectively. If two observations are available for each unique reflection (which may, for example, happen when the data collection covers the asymmetric unit of reciprocal space two times in a row), then ∊_1_ and ∊_2_ are (negatively) correlated, but τ is not correlated with ∊_1_ or ∊_2_.

In all cases of systematic error we can still assume that *E*(∊_1_τ) = *E*(∊_2_τ), since the random assignment of measurements to half-data sets on average prevents any particular of the two expectation values being larger than the other.

The difference CC*^2^ – CC^2^_true_ is zero if the above assumptions about the mutual independence of τ, ∊_1_ and ∊_2_ hold. It is interesting that even after dropping these assumptions we can calculate CC*^2^ − CC^2^_true_. This offers a way to predict whether the estimate CC* overestimates or underestimates CC_true_. Collecting and rearranging terms as in the Supplementary Material of Karplus & Diederichs (2012 ▶), we obtain
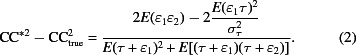
The denominator of the right-hand side is positive. It is noteworthy that no matter whether the true signal τ is negatively or positively correlated with ∊_1_ and ∊_2_, the second term of the numerator is always negative. Overall, the numerator may be positive or negative, or its two terms could cancel. Cases where ∊_1_ and ∊_2_ are positively correlated, as in the first two of the three examples above, seem to have practical relevance; for these, the first term *E*(∊_1_∊_2_) is larger than zero, which favours CC* > CC_true_ (*i.e.* overestimation of CC_true_). However, the second term may cancel the first term, or if it is larger than the first term the overall result may be an underestimation of CC_true_.

The third example yields CC*^2^ − CC^2^_true_ < 0, which means that CC* is an underestimate of CC_true_. The deviation from the truth occurs in opposite directions in the two half data sets, so that their average is close to the truth (high CC_true_) but their agreement may be poor (low CC_1/2_) such that CC* < CC_true_.

These examples demonstrate that even if the assumption that CC* is an accurate estimate of CC_true_ may not be completely fulfilled, CC* is often a conservative estimate of CC_true_ owing to the safeguarding effect of the second term of the numerator. This clearly is a desirable property of a data-quality indicator.

We also note that in the case of vanishing signal (τ→0) only the first term of the numerator remains. Thus, at high resolution the value of CC*^2^ − CC^2^_true_ is dominated by the expectation value of ∊_1_∊_2_.

### 3.4. Application of the theory to the specific data-filtering test cases

Applying these theoretical considerations to the two data-filtering practices explored, we can understand that the decreases in CC_1/2_ do not in this case occur owing to there being less signal in these data. Rather, the reduction of CC_1/2_ is owing to systematic error being introduced: if negative unique reflections are rejected (data set CDO3b) then in high-resolution shells where the signal vanishes, ∊_1_ and ∊_2_ become negatively correlated (Fig. 3 ▶ *b*). This, according to (2), is augmented by the correlation between τ and ∊_1_, ∊_2_, leading to a substantial reduction of CC* below CC_true_. In other words, the systematic error causes CC_1/2_ to be an underestimate of the level of signal in the data, meaning that in turn CC* is no longer a valid upper limit for CC_work_ and CC_free_. Indeed, consideration of the refinement results demonstrates that at high resolution CC_work_ and CC_free_ are both higher than CC* for data set CDO3b (Table 4 ▶).

Considering data set CDO3c, the CC_1/2_ (and thus the CC*) value is lower than that of CDO3, but to a lesser extent than CDO3b (Fig. 2 ▶). Since rejection of negative observations does not necessarily result in a correlation between ∊_1_ and ∊_2_ (Fig. 3 ▶ *c*), the reason for the decrease is not the first term of the numerator, as for CDO3b, but rather the second term. Here, the rejection of negative observations increases the intensity of the merged data over that of the true data, which leads to a correlation between τ and ∊_1_, ∊_2_. Again, according to (2), this decreases CC* below CC_true_. The fact that CC_work_ and CC_free_ are much reduced for CDO3c compared with models refined against CDO3 or CDO3b (Table 4 ▶) is owing to the fact that at high resolution the rejection of negative observations leads to systematically increased intensities for the affected reflections, but not for reflections without negative observations. This is a systematic error that the model cannot fit; *i.e.* it reduces CC_work_ and CC_free_. This effect does not happen for CDO3b, which just has its weak reflections discarded; in this latter case, CC_work_ and CC_free_ are higher than for the model refined against CDO3 since the model refined against CDO3b fits the subset of stronger, less noisy intensities.

The rejection of negative intensities in this section serves as an example for a broader class of data-massaging practices, namely all those employing a positive σ cutoff. Employing such a cutoff can be expected to result in even worse models than those obtained with a cutoff of zero, as shown here. Negative σ cutoffs of about −3σ and below, on the other hand, may be expected to reject true outliers and to affect very few reflections. In addition, it should be noted that the artificial increase of average intensities at high resolution brought about by rejection of weak reflections invalidates the French–Wilson procedure for estimating amplitudes from intensities and may result in a model with unrealistically low temperature factors.

Unfortunately, non-isomorphism between data sets cannot be treated using the theory laid out above, since the concept of ‘truth’ is undefined when two data sets from different crystals are merged: if the data sets are best described by different structures, then which is the ‘true’ one? Obviously, further research is needed to obtain meaningful prediction of the model quality resulting from the merging of slightly non-isomorphous data sets (Giordano *et al.*, 2012 ▶).

## 4. Summary

Since the introduction of data *R* values, decisions based on these have been influencing protocols dealing with rejection of complete data sets, resolution shells with weak data and weak (*e.g.* negative) reflections. Procedures such as those analyzed here that discard, filter or massage data in order to minimize data *R* values need to be abandoned since they lead to suboptimal atomic models. They should be replaced by evaluations of data quality using better suited correlation-coefficient-based criteria, together with unambiguous identification of the best models, which can be performed using a paired-refinement strategy.

## Figures and Tables

**Table 1:** Statistics of single and merged CDO data sets The resolution range is 50–1.57 Å; values in parentheses are for the highest shell (1.61–1.57 Å). CC statistics are only given for the highest resolution shell because the overall CC values are always close to 1 and thus are uninformative. For CC_1/2_, CC* and CC_work_ (based upon ∼2000 reflection pairs per shell) the values in the lower resolution shells are always higher than those in the highest resolution shell. This is not always true for CC_free_, which owing to the smaller set of reflections (∼100 reflection pairs in each shell) has a standard error (∼0.1) that is much larger than that of CC_work_ (∼0.02).

Data-set name	CDO3	CDO4	CDO5	CDO3+4	CDO3+5	CDO4+5	CDO3+4+5
Data processing							
No. of observations	201160 (10837)	155771 (2389)	200117 (10838)	358117 (13657)	401270 (21717)	357787 (13655)	558273 (24518)
No. of unique reflections	29424 (2008)	26807 (1316)	27939 (1982)	29431 (2013)	29433 (2013)	28195 (1995)	29433 (2013)
Completeness (%)	99.9 (98.7)	93.8 (79.3)	95.7 (98.0)	99.9 (98.8)	99.9 (98.8)	95.7 (98.0)	99.9 (98.8)
*R*_meas_ (%)	10.2 (294.0)	23.1 (431.1)	26.0 (395.6)	15.0 (314.7)	15.9 (332.6)	26.0 (401.4)	15.9 (339.8)
〈*I*/σ〉	16.24 (0.64)	10.68 (0.21)	9.88 (0.19)	13.63 (0.69)	14.12 (0.87)	13.76 (0.49)	14.60 (0.91)
CC_1/2_ in highest shell; No. of pairs	0.208; 1986	0.058; 842	0.127; 1961	0.175; 2006	0.223; 2008	0.154; 1992	0.222; 2008
CC* in highest shell[Disp-formula fd1]	0.587	0.331	0.475	0.546	0.603	0.517	0.602
Isotropic refinement							
Highest shell CC_work_, CC_free_	0.541, 0.581	0.256, 0.131	0.383, 0.425	0.522, 0.487	0.529, 0.596	0.432, 0.385	0.536, 0.526
Overall *R*_work_, *R*_free_	0.186, 0.219	0.211, 0.252	0.198, 0.236	0.185, 0.221	0.185, 0.216	0.199, 0.237	0.186, 0.221
R.m.s.d. from ideality: bonds (Å)/angles (°)	0.015/1.57	0.016/1.53	0.016/1.51	0.015/1.55	0.015/1.53	0.015/1.51	0.015/1.54

**Table 2:** Results (*R*_work_, *R*_free_) of pairwise refinements Within each row of the table, the same sets of reflections are used. Values in parentheses are copied from Table 1 ▶; values in bold denote improvements in *R*_free_ of models refined against a merged data set, compared with models refined against the single data set.

	Model refined against			
Data set	CDO3+4	CDO3+5	CDO4+5	CDO3+4+5
CDO3	0.188, 0.220	**0.189**, **0.218**	Not determined	0.192, 0.221
CDO4	0.227, 0.262	Not determined	0.215, 0.253	0.224, 0.257
CDO5	Not determined	0.215, 0.243	**0.200**, **0.234**	0.211, 0.240
CDO3+4	(0.185, 0.221)	Not determined	Not determined	0.186, **0.219**
CDO3+5	Not determined	(**0.185**, **0.216**)	Not determined	0.186, 0.219
CDO4+5	Not determined	Not determined	(**0.199**, **0.237**)	0.211, 0.244

**Table 3:** Application of the pairwise refinement technique to the data sets specified Within each row of the table, the same sets of reflections are used to calculate *R*_work_ and *R*_free_. Each model (top row) was obtained by refinement against one data set. Its model *R* values (*R*_work_, *R*_free_) against the other data sets are also given. For each data set, the model that gives the best *R*_free_ is marked in bold.

*R* factors calculated against	Model refined against		
	CDO3	CDO3b	CDO3c
CDO3 (all)	**0.186**, **0.219**	0.187, 0.223	0.187, 0.227
CDO3b (positive unique)	**0.178**, **0.211**	0.178, 0.216	0.180, 0.220
CDO3c (positive observations)	**0.204**, **0.233**	0.204, 0.235	0.199, 0.239

**Table 4:** Comparison of CC* with CC_work_, CC_free_ in the highest resolution shell (1.61–1.57 Å) All of the data from CDO3 were used or only positive unique reflections or only positive observations were used. The number of unique reflections is given in parentheses.

	Model refined against		
	All reflections (CDO3)	Positive unique reflections only (CDO3b)	Positive observations only (CDO3c)
CC*	0.587	0.174	0.477
CC_work_, CC_free_	0.540 (1912), 0.581 (99)	0.580 (1308), 0.612 (73)	0.385 (1872), 0.403 (94)
